# Emerging Roles of Exosomes in Huntington’s Disease

**DOI:** 10.3390/ijms22084085

**Published:** 2021-04-15

**Authors:** Hanadi Ananbeh, Petr Vodicka, Helena Kupcova Skalnikova

**Affiliations:** Institute of Animal Physiology and Genetics of the Czech Academy of Sciences, Laboratory of Applied Proteome Analyses and Research Center PIGMOD, Rumburska 89, 277 21 Libechov, Czech Republic; ananbeh@iapg.cas.cz (H.A.); vodicka@iapg.cas.cz (P.V.)

**Keywords:** extracellular vesicle, exosome, neurodegeneration, Huntington’s disease, huntingtin, polyQ, biomarker, therapy

## Abstract

Huntington’s disease (HD) is a rare hereditary autosomal dominant neurodegenerative disorder, which is caused by expression of mutant huntingtin protein (mHTT) with an abnormal number of glutamine repeats in its N terminus, and characterized by intracellular mHTT aggregates (inclusions) in the brain. Exosomes are small extracellular vesicles that are secreted generally by all cell types and can be isolated from almost all body fluids such as blood, urine, saliva, and cerebrospinal fluid. Exosomes may participate in the spreading of toxic misfolded proteins across the central nervous system in neurodegenerative diseases. In HD, such propagation of mHTT was observed both in vitro and in vivo. On the other hand, exosomes might carry molecules with neuroprotective effects. In addition, due to their capability to cross blood-brain barrier, exosomes hold great potential as sources of biomarkers available from periphery or carriers of therapeutics into the central nervous system. In this review, we discuss the emerging roles of exosomes in HD pathogenesis, diagnosis, and therapy.

## 1. Introduction

**Figure 1:**
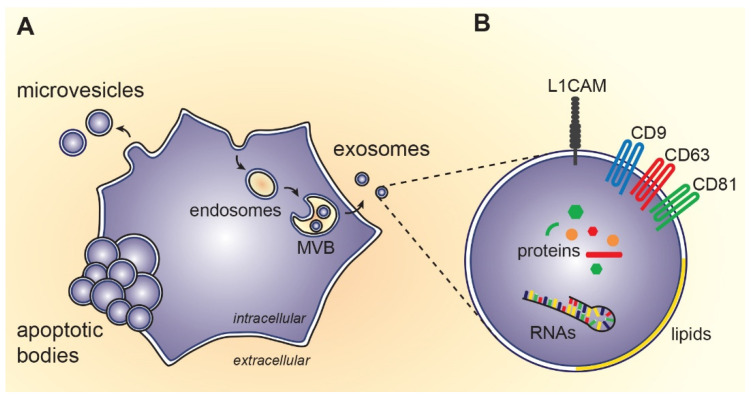
Biosynthesis and composition of extracellular vesicles: (**A**) Exosomes originate from endosomal compartment, i.e., intraluminal vesicles of multivesicular bodies (MVB). On the other hand, microvesicles arise by outward budding of cytoplasmic membrane and apoptotic bodies are formed during programmed cell death; (**B**) Exosomes contain proteins, RNAs, lipids and small molecules of the source cell. Some of the molecules specifically incorporated during exosome biosynthesis can be used as markers for exosome detection (e.g., transmembrane tetraspanins CD9, CD63, CD81) or as a marker of neuronal origin (e.g., L1 cell adhesion molecule, L1CAM).

Extracellular vesicles (EVs) are phospholipid bilayer membrane enveloped particles released from cells into extracellular environments and body fluids. According to the mechanism of biogenesis, three main EV subtypes can be distinguished, i.e., exosomes, microvesicles, and apoptotic bodies (Figure 1) [1,2]. This review is focused mainly on exosomes, as they are the most studied vesicle subtype in human diseases, including Huntington’s disease. However, the original terminology of EV labeling by the authors of cited literature is respected in our review.

Exosomes are nano sized EVs (40–120 nm) that were initially identified as a part of reticulocyte maturation in sheep [3]. Exosomes were later observed to be released by almost all cell types [4]. Exosomes play an important role in cell-cell communication and intercellular signaling by transferring molecules into recipient cells [5,6,7]. Due to their roles in the physiological and pathological conditions in the brain, their ability to cross the blood-brain barrier and their cargo, exosomes are a potential source of biomarkers or therapy carriers in neurodegenerative diseases (NDs).

## 2. Exosome Biogenesis

Exosomes are formed by endocytosis and are released by exocytosis (Figure 1). Inward budding of the plasma membrane results in the formation of small intracellular vesicles which then fuse together and form early endosomes [8]. The invagination of early endosome membranes results in the formation of intraluminal vesicles (ILVs) within large multivesicular bodies (MVBs). During the maturation of early endosomes to late endosomes, some proteins are directly integrated into the invaginating membrane, whilst the cytoplasmic molecules such as proteins, messenger RNAs (mRNAs), and microRNAs (miRNAs) are engulfed and enclosed in the ILV lumen. Some MVBs fuse with the plasma membrane and release their contents, the exosomes, into the extracellular space. Other MVBs are delivered to lysosomes for degradation [9,10,11]. Exosome biogenesis depends on the endosomal sorting complexes required for transport (ESCRTs), which recognize ubiquitinated proteins [12]. ESCRTs consist of four protein complexes ESCRT-0, ESCRT-I and ESCRT-II, and ESCRT-III that work cooperatively to facilitate the MVB formation, vesicle budding, and protein cargo sorting [9,11,13,14].

## 3. Content of Exosomes

The biological cargo of exosomes varies widely depending on their cell type of origin and their activation sites [15]. They mainly consist of lipids, proteins, nucleic acids, specifically mRNAs and miRNAs, and small molecules [16,17]. More than 4000 different proteins and over 2400 different RNAs have been identified and characterized in exosomes [18].

Owing the endosomal origin, exosomes are enriched in several proteins that are engaged in the biogenesis of MVBs, such as clathrin, ALIX (ALG-2 interacting protein X), TSG101 (tumor susceptibility gene 101) and ubiquitin [19]. In addition, exosomes contain CD9, CD63, CD81, CD82, CD54 and CD11b tetraspanins that serve as distinctive markers [20,21]. Moreover, heat shock proteins (HSP90, HSP70, HSP60) are common exosomal proteins that act as chaperones and play an essential role in cellular responses related to the environmental stress [19]. There are other exosome enriched molecules, including signal transduction molecules (i.e., ADP-ribosylation factor, cell division control protein 42, epidermal growth factor receptor, etc.) and lipid raft-related molecules, such as flotillin, phosphatidylserine, and sphingomyelin [22]. Exosomes contain cytoplasmic proteins (i.e., annexins), enzymes (i.e., protein kinase C, glyceraldehyde 3-phosphate dehydrogenase, etc.), and, depending on exosome origin, immunoregulatory molecules such as the major histocompatibility molecules (MHC) class I and II that have a significant role in immunoregulation by presenting antigenic peptides [19,23].

Exosomes carry functional RNA and miRNA that can be transferred into recipient cells and induce cellular response [7]. In contrast to cells, exosomes contain very little or no ribosomal RNA [24]. RNAs and miRNAs enveloped inside the extracellular vesicles are protected from nucleases, therefore they may have long lasting effects on disease related gene expression. In addition, selected exosomal miRNAs have specific profiles in different central nervous system (CNS) disorders even in the presymptomatic stages and their expression significantly changes under the neuropathological conditions, which make them promising biomarkers [25,26,27].

## 4. Huntington’s Disease

Huntington’s disease (HD) is a rare, progressive, and incurable neurodegenerative disorder with age of onset between 35 and 45 years and death occurring 15 to 17 years after onset [28,29,30]. The prevalence of HD is 5.7 persons per 100,000 worldwide with an average of 10.6 to 13.7 individuals per 100,000 in the European population [30,31,32]. In certain regions (i.e., Australia, North America, and Western Europe including the United Kingdom), the prevalence of the disease has increased over the past 50 years [33]. HD is characterized by progressive movement disorder (chorea, saccadic eye movement abnormalities, ataxia of speech, dysphagia, etc.), cognitive dysfunction (dementia), and psychiatric disturbances (depression, anxiety, apathy, etc.) [29,30]. Progressive movement failure is the main reason for life-ending complications.

HD has autosomal dominant heredity and is caused by an expanded Cytosine-Adenine-Guanine (CAG) repeat (≥36) in the huntingtin coding gene (IT15) on chromosome 4, resulting in the expression of mutant huntingtin protein (mHTT) with abnormal number of glutamine repeats (polyQ) in its N terminus [32,34]. The CAG repeats below 26 (common range in humans: 17–25) are considered normal and do not cause the disease. The intermediate range repeats (27 to 35) mostly do not cause HD with a few reported exceptions [35]. The CAG repeat range of 36–39 might be found in affected individuals as well as asymptomatic individuals (reduced penetrance alleles), whereas individuals with ≥40 repeats always develop the disorder (fully penetrant alleles) [32]. The number of CAG repeats is inversely correlated with the age of onset, longer repeats predict earlier onset of the disease and vice versa [36,37]. Predictive and diagnostic genetic testing is available to detect the expanded CAG repeats for the affected individuals as well as for individuals at risk [38]. Pretest counselling is essential to consider the test result impact on the patient and family [39].

There is no therapy available to slow down the disease progression and the HD treatment is largely focused on management of symptoms to control the motor and psychiatric disturbances [40,41]. Recently, new therapies targeting either DNA or RNA of mHTT to reduce the mHTT expression are being developed, using antisense oligonucleotides (ASOs), RNA interference (RNAi), zinc finger proteins (ZFPs), and the CRISPR-Cas9. Such huntingtin lowering strategies are considered the most promising treatments as they are targeting the proximate cause of the disease (Table 1) [32,41,42,43,44,45]. Over the past decades, clinical trials targeting the disease modification and symptomatic treatments in HD have begun, a number of these trials have failed and vast number are currently ongoing [46]. Availability of biomarkers, particularly accessible with minimal invasivity, is essential to estimate the treatment efficiency [47].

### 4.1. Huntingtin Protein

**Figure 2:**
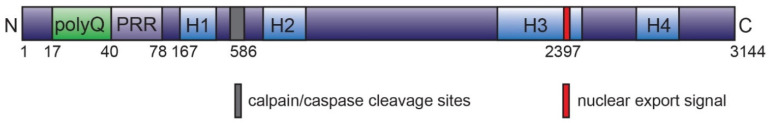
Overview of human huntingtin protein. Full-length HTT consists of 3144 amino acids, with a total molecular mass of 350 kDa. The N-terminal 17 amino acid region carries several post translational modification sites (such as acetylation, SUMOylation, phosphorylation, ubiquitination, etc.) and is followed by polyglutamine tract (polyQ; expanded in Huntington’s disease), proline rich region (PRR), HEAT repeat domains (H1-H4; the HEAT corresponds to proteins, where they were first described: huntingtin, elongation factor 3 [EF3], protein phosphatase 2A [PP2A], and the yeast kinase TOR). Leucine-rich nuclear export signal is located at the C-terminal region. The protein may undergo post translational modifications and/or protease cleavage on several sites, e.g., caspase-6 cleavage at aspartate 586 [50,72,75,76,77].

Huntingtin (HTT) is a large soluble protein (350 kDa), consisting of 3114 amino acids. The HTT protein is characterized by the presence of polyglutamine (polyQ) region, a proline rich region (PRR), and HEAT repeats (Huntingtin, Elongation factor 3, protein phosphatase 2A, Target of rapamycin 1) that are important for protein interactions, and caspase and calpain cleavage sites in higher vertebrates (Figure 2) [48,49]. The N-terminal 17 amino acids (N17) have been identified as a critical region that has a role in HTT localization, aggregation, and toxicity, and it is the subject of several post translational modifications including acetylation, SUMOylation, phosphorylation, and ubiquitination [49,50].

PRR is found only in mammals, is variable in the non-HD population, and is essential for HTT interactions with proteins that contain tryptophanes or Src homology 3 domains. PRR deletion has no extreme effects on mouse behavior [67]. The PRR has a proline-proline helix which might be important for stability of the polyQ structure and the tendency of mHTT to aggregate [67]. HEAT repeats consist of around 50 amino acids and contain two antiparallel α-helices forming a hairpin that normally acts as scaffold for various protein complexes and mediates inter and intra molecular interactions [67,68]. A total of 16 HEAT repeats has been identified in the HTT protein and they are organized into 4 clusters [68] (Figure 2). Several proteolytic cleavage sites including proline, glutamic acid, serine, and threonine domains have been identified in HTT. These domains are found on both wild type and mHTT [67,69]. Proteases that are responsible for HTT cleavage include: caspases, calpain, cathepsins, aspartic endopeptidases, and the metalloproteinases [70,71]. HTT is cleaved by caspase 3 at amino acids 513 and 552, caspase 1 at position 572, caspase 2 at amino acid 552, and caspase 6 at amino acid 586 [72,73,74]. The two calpain cleavage sites are located at amino acids 469 and 536, and the MMP-10 metalloproteinase cleaves HTT at amino acid 402 [67,68].

HTT is ubiquitously expressed in the human tissues and organs with higher expression in the CNS and testes. HTT is essential for normal embryonic development [75], as HTT gene knockout is lethal in mice by 8.5 embryonic day [78,79]. HTT is engaged in many cellular and biological functions such as transcription, transport, vesicular trafficking, coordination of cell division, energy metabolism and antiapoptotic activity [42,49], and co-localizes with many organelles such as the nucleus, endoplasmic reticulum, Golgi complex, endosomes, mitochondria, and synaptic vesicles [48,80]. This might reflect its role as a scaffold protein that is engaged in many protein-protein interactions and the formation of multi-protein complexes [81]. Despite decades of research, the HTT roles in cells are not yet fully understood.

### 4.2. Pathogenesis of Huntington’s Disease

In general, the aggregation of misfolded proteins is the distinctive characteristic of neurodegenerative diseases (NDs) including Alzheimer’s disease (AD), Parkinson’s disease (PD), Amyotrophic lateral sclerosis (ALS), HD, and others. These protein aggregates form different types of inclusions, such as amyloid β and tau in AD, α-synuclein in PD, superoxide dismutase 1 in ALS or mHTT aggregates in HD.

**Figure 3:**
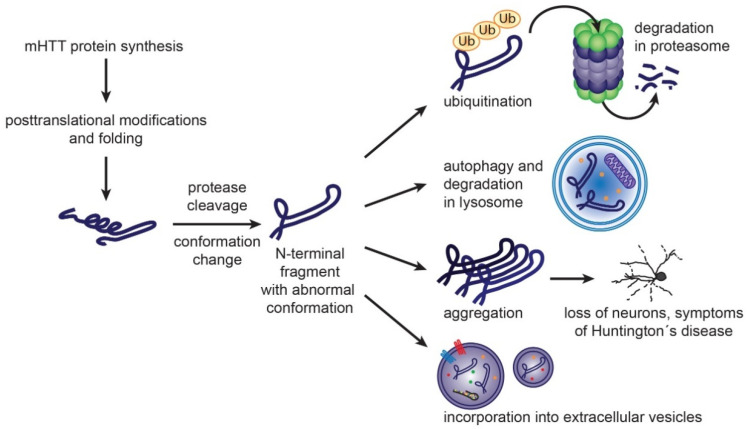
Formation of mHTT toxic species and cellular mechanisms to handle them. Mutant huntingtin protein carrying expanded polyglutamine sequence is prone to adopt abnormal conformation. Post translational modifications and protease cleavage may contribute to formation of misfolded N-terminal fragments. Molecular chaperones, such as Heat Shock Proteins, participate in re-folding of misfolded proteins. Once unsuccessful, the resulting misfolded proteins can be degraded by ubiquitin-proteasome system or autophagy. Accumulation of misfolded mHTT rich in β-sheet structure leads to aggregate formation, loss of neurons and Huntington’s disease symptoms. Misfolded proteins may be eliminated from neurons also by exosomal secretion [86,87,88].

Despite that the gene mutation causing HD was discovered in 1993 [76], the HD pathogenesis is not fully elucidated yet. The expanded polyQ sequence in mHTT protein has tendency to undergo conformational changes to form β-pleated sheet prone to aggregation [77,82]. The early phases of aggregate formation appear to be accelerated by hydrophobic interactions with an amphipathic α-helical structure of N17 [83,84]. Molecular chaperones play a major role in mHTT protein folding and re-folding [85]. Misfolded mHTT or its fragments and oligomers may undergo degradation in ubiquitin-proteasome system (UPS) or by autophagy (Figure 3).

Under physiological conditions, proteostasis ensures balance between protein synthesis, folding, trafficking and degradation. The impairment of the proteostasis systems aggravates the aggregation of the misfolded huntingtin. Efficacy of proteostasis mechanisms declines with age. Experimental manipulation of distinct proteostasis nodes, such as molecular chaperones, UPS or autophagy may reduce toxic mHTT aggregate formation [81,85].

Post translational modifications also influence the mHTT toxicity, aggregation propensity and intracellular localization. Proteolytic cleavage of mHTT generates N-terminal fragments with increased tendency to aggregate. Such shorter fragments may be also formed by aberrant splicing of HTT coding mRNA [45]. Nuclear localization of mHTT increases its toxicity. On the other hand, HTT phosphorylations at Thr3 and Ser13 and/or Ser16 are potentially protective against aggregation [77]. Ubiquitination, acetylation and SUMoylation target the mHTT protein for degradation via ubiquitin proteasome system or autophagy [34].

The mHTT inclusions can induce a physical block of axonal transport between the cell body and the synaptic cleft and recruit other polyQ-containing proteins (i.e., transcription factors), which then interact with mHTT and might lose their physiological functions, leading to cell death [89,90]. The mHTT inclusions might thus function as sinks where vital proteins are sequestrated, compromising cell survival [91].

## 5. Exosomes in Huntington’s Disease

**Figure 4:**
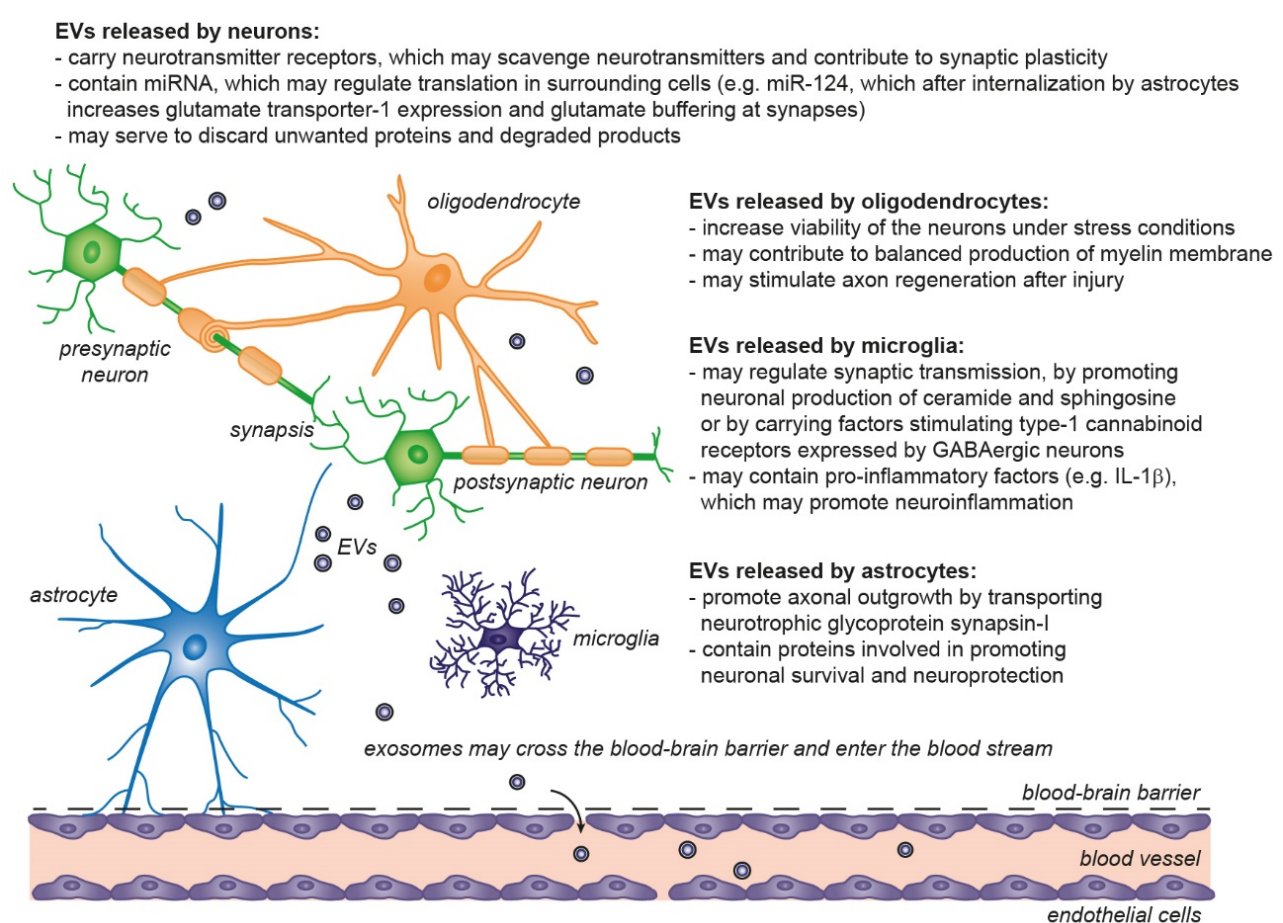
Physiological roles of extracellular vesicles in the central nervous system. EVs released from neurons and glial cells show neurotrophic and neuroprotective effects, by regulation of synaptic transmission/plasticity, protection against stress and excitotoxicity and may support axon regeneration after injury. Astrocytes and microglia participate in removal of EVs produced by other cells and may contribute to degradation of unwanted proteins (including unfolded proteins) released through EVs [86,87,88,105,106,107].

In the CNS, exosomes play essential physiological roles in the cell to cell communication and homeostasis maintenance required for normal brain function [92], particularly in neurogenesis [92,93,94,95], in synaptic activity and plasticity [96], myelination [88], and protection and regeneration of neuron after injury and disease [97,98,99] (Figure 4). EVs are released by neural cells including neurons, astrocytes, microglia, and oligodendrocytes [92,93,100] under both normal and pathological conditions. In human CNS, EVs have been isolated from the cerebrospinal fluid (CSF) and from adult brain [101,102]. The cellular origin of the neuronal secreted exosomes can be assessed by the presence of the GPI-anchored prion protein, cell adhesion molecule L1, and subunits of glutamate receptors [103,104].

### 5.1. Exosomes in Misfolded Protein Spreading

Recently, exosomes have emerged as a common player being able to mediate the pathogenesis of neurodegenerative disorders [108]. It has become clear that proteins related to NDs and prion diseases can be selectively integrated into ILVs of MVBs and subsequently released into the extracellular environment within exosomes [95].

Several studies showed that the neurotoxic misfolded proteins in NDs such as prion protein, α-synuclein, amyloid precursor protein, and tau, are transferred across the CNS by exosomes contributing to the spread and progression of prion disease and amyloidogenesis [16].

In HD, spreading of mHTT between cells is evident mainly from in vitro and animal experiments that revealed internalization of synthetic peptide (44Q) into cells and formation of cytoplasmic aggregates [109], as well as mHTT aggregate transmission between cells [110,111,112,113] and mHTT propagation between neurons [114]. Interestingly, the possibility of the mHTT spreading into genetically unrelated tissue was confirmed in vivo in 3 HD patients undergoing fetal striatal tissue transplantation, where mHTT aggregates were formed in the transplanted tissue [115].

Exosomes are supposed to be involved in such mHTT protein propagation between cells, both on protein and RNA levels. The injection of exosomes released from fibroblasts of HD patients into a newborn mouse brain’s ventricles triggered the manifestation of HD-related behavior and pathology [116]. However, this finding needs further confirmation, as other factors could co-isolate with exosomes from fibroblast conditioned medium by ExoQuick precipitation. At the RNA level, the ability of EVs to deliver toxic expanded trinucleotide repeat RNAs from one cell to another was reported in human 293T cell culture [117]. On the other hand, neuroprotective roles of exosomes secreted from astrocytes and adipose tissue-derived stem cells have been reported. Injection of astrocytic exosomes into the striatum of HD 140Q knock-in (KI) mice reduced the density of mHTT aggregates. Interestingly, presence of mHTT protein in exosomes released by primary astrocytes from 140Q KI mice was not detected, suggesting a potential use of astrocyte-derived exosomes in HD therapy [15]. Similarly, exosomes secreted from adipose-derived stem cells, that are known to release neurotrophic factors, were shown to reduce the mHTT aggregates, mitochondrial dysfunction, and cell apoptosis of in vitro HD model [118]. Interestingly, neuroprotective synaptic chaperone cysteine string protein α (CSPα) was observed to support the export of polyQ expanded (72Q) huntingtin exon 1 from cells via EVs. The CSPα may thus participate in protein homeostasis maintenance at synapses and mHTT clearance from cells [119].

### 5.2. Diagnostic Potential of Exosomes

Exosomes have large potential as non-invasive diagnostic biomarkers’ carriers for NDs as well as many other diseases. There are several reasons that make them attractive targets for clinical diagnostics and biomarker discovery. First, exosomal contents (lipids, proteins, nucleic acids, etc.) are changing during disease and might reflect the disease progress. Second, exosomes can be isolated non-invasively from easily accessible biological fluids including blood, urine, and saliva. The non-invasive availability is very important for early disease diagnosis especially in NDs. Third, exosomes have a double layer membrane, which protects the potential biomarkers from degradation. Fourth, exosomes are highly stable, making their clinical use practical, as samples can be kept for a long period before analysis. Fifth, exosomes carry markers related to their cellular origin, therefore, the origin can be traced. Finally, exosomes can pass through the blood-brain barrier from blood to brain and vice versa, providing information about nervous cells that is hard to obtain without the use of invasive techniques [12,98,120]. Many studies reported exosomes being biomarker sources for various NDs including AD [104,121,122,123,124], PD [125,126,127,128,129], ALS [118], and prion diseases [95].

Reliable non-invasive biomarkers reflecting disease progression are highly important for patients to provide early diagnostic proof or enable monitoring of currently emerging therapies [26]. Misfolded proteins or protein aggregates/inclusions are the pathological hallmark of many neurodegenerative disorders including HD, therefore the study of these proteins might be essential in developing new biomarkers [118]. Exosomes might contain the mHTT, its fragments, or other proteins reflecting the conditions of exosomes producing CNS cells. Such proteins might serve as potential biomarkers of HD. To date, there are only few studies that analyzed exosomes or their composition in the search for HD biomarkers, and more investigations are in need.

Blood, as the biological fluid easily collectable with minimal invasiveness, contains extracellular vesicles from the whole organism, with the majority (70–90%) of vesicles originating from platelets [130,131]. Thus, the platelets and platelet-derived EVs were searched as potential HD biomarker carriers. However, no differences were found in the number of EVs released by platelets between HD patients and controls and no correlations of platelet-derived EV numbers with age, CAG repeat number and disease stage were observed [132]. Platelets from HD patients contain highest amounts of mHTT among all blood cells [133]. Surprisingly, in EVs derived from platelets, the mHTT protein was undetectable, even after platelet activation [132]. In this study, the platelet-derived EVs were pelleted by centrifugation at 20,000× *g* for 90 min and the mHTT was detected in EV lysates by highly sensitive 2B7-MW1 Singulex detection assay (sensitivity 4 pg/mL) [132]. Altogether, platelet-derived EVs do not seem to be valuable biomarkers of HD.

Application of omics techniques enabled to map the molecular composition of EV in NDs. While EV protein and miRNA alterations have been studied in more frequent Alzheimer’s and Parkinson’s diseases [25,134], only little is known in HD.

Proteomic analysis of urinary EVs in more than 100 participants was performed by Wang et al., and revealed the enrichment of endolysosomal proteins linked to PD, AD, and HD, which makes the urinary EVs a highly accessible resource for biomarker discovery with particular promise for NDs [135].

Roles of miRNAs in disease pathophysiology are extensively studied as miRNA are significant regulators of gene expression. Several exosomal miRNAs are candidates for ND biomarkers, such as decreased miR-342-3p, miR-125a-5p, miR-125b-5p, miR451a, miR-23a-3p, and miR-126-3p, in AD [136,137,138] or increased miR-331-5p, miR-22*, miR-23a, miR-24, in PD [138,139]. However, in Huntington’s disease, the miRNA content in exosomes was not studied yet. Reed et al. identified 6 significantly increased miRNAs (miR-135b-3p, miR-520f-3p, miR-4317, miR-3928-5p, miR-140-5p, and miR-8082) in CSF in the prodromal HD gene polyQ expansion carriers compared to controls [140]. In blood plasma of symptomatic HD patients, 13 miRNAs (miR-877-5p, miR-223-3p, miR-223-5p, miR-30d-5p, miR-128, miR-22-5p, miR-222-3p, miR-338-3p, miR-130b-3p, miR-425-5p, miR-628-3p, miR-361-5p, miR-942) were upregulated [141]. Several studies reported a decrease of miR-124 expression in HD patients’ brains [142,143], STHdh*^Q111^*/Hdh*^Q111^* HD cell line, and R6/2 HD mouse [144]. MiR-124 plays a key role in neurogenesis and is the most abundant miRNA in the adult brain [145]. Detection of miR-124 in body fluids, whether in free-form or packed into exosomes, and its possible association with HD, deserve further studies.

### 5.3. Exosomes in Delivery of Therapeutics

EVs, due to their ability to cross blood-brain barrier [146] and biocompatibility, are promising therapeutic drug carriers into the CNS in NDs with few ongoing preclinical trials in AD and PD [147,148,149]. In HD, exosomes are particularly efficient in delivery of oligonucleotide therapeutics (miRNA and siRNA) (Table 1). Oligonucleotide therapeutics are a novel class of drugs targeting RNA or DNA to prevent expression of the protein responsible for the disease phenotype [54] and primarily include miRNAs, siRNAs and ASOs.

Recently, miRNAs-based therapy has emerged as a promising technique in NDs’ treatments [150]. miR-124 was selected in the majority of exosome-based miRNA delivery studies for ND treatment because it is highly and specifically expressed in all brain regions except for the pituitary gland, and at 100 times lower expression in other tissues, and has a regulatory role in CNS development and diseases [151]. miR-124 supports adult neurogenesis and expression of brain-derived neurotrophic factor by downregulating the expression of repressor RE1-Silencing Transcription Factor (REST) [57,152] and is one of the most downregulated miRNAs in HD. It has been reported that miR-124 slows down the progression of HD in R6/2 HD transgenic mouse through promoting neuronal differentiation and survival [153]. In 2017, Lee et al. developed an exosome based delivery to mouse HD model by generating miR-124 expressing HEK293 cell line to produce the exosomal miR-124 [57]. Such exosomes were injected into the striatum of R6/2 transgenic HD mice which resulted in reduction of REST protein expression. Unfortunately, no significant improvement in rotarod performance was observed in this study one week after miR-124 delivery, and longer time intervals have not been studied [57]. Due to the mild therapeutic efficiency of exosomal miR-124, Lee et al. suggested the increment of miRNAs’ dose packed in the exosomes, and also suggested the use of other miRNAs (i.e., miR-9, miR-22, miR-125b, miR-146a, miR-150, and miR-214) to be delivered by exosomes as they might have greater therapeutic impacts than miR-124 [57].

A small interfering RNA (siRNA) binds a target mRNA, guiding mRNA cleavage through RNA-induced silencing complex (RISC) which can provide effective long-term gene silencing [53,54]. The therapeutic potential of exosome-mediated siRNA delivery in BACHD and N171-82Q mice models was tested by Wu et al. [56]. Modified exosomes expressing the neuron-specific rabies viral glycoprotein (RVG) peptide loaded with siRNA targeting human huntingtin exon 1 (HuHtt) transcript was used. Then, HuHtt-siRNA RVG exosomes were injected intravenously to normal mice, and BACHD and N171-82Q transgenic mice at 10 mg/kg every two days for two weeks. siRNA was efficiently delivered into the mouse brain by RVG-modified exosomes and HuHtt-siRNA RVG exosomes significantly reduced Htt expression up to 46% and 54%, respectively, in transgenic mouse lines. N17-82Q mice receiving RVG exosomes showed improvement on the Rotarod performance. The study indicated the therapeutic potential of HuHtt-siRNA RVG exosomes in HD [56].

Hydrophobically-modified small interfering RNA (hsiRNA) are asymmetric oligonucleotides with chemical modifications that enhance stability and promote cellular internalization. They efficiently bind cellular membranes, enter cells, and stimulate gene silencing [53,54,154]. Exosomes are promising natural nano-devices for therapeutic RNA delivery but loading of the sufficient dose of RNAs remains a challenge [55]. To increase the efficiency, Bicans et al. synthesized a group of cholesterol-conjugated hsiRNAs to be loaded into exosomes. Cholesterol-conjugated hsiRNAs delivered by exosomes (100,000 g fraction) were more effective at inducing HTT mRNA silencing in neurons compared to cholesterol hsiRNA alone. In addition, cholesterol hsiRNAs did not induce the silencing of the target mRNA when delivered by large EVs (10,000 g fraction) [55].

In another study with hsiRNA targeting huntingtin RNA, exosomes were loaded by hsiRNA by simple co-incubation. In in vitro experiments, such exosomes mediated internalization of hsiRNA^HTT^ into primary cortical neurons leading to dose-dependent silencing of Htt mRNA and protein in these cells. Unilateral infusion of such hsiRNA^HTT^- loaded exosomes into mouse striatum resulted in bilateral hsiRNA^HTT^ distribution in striatal and cortical regions and statistically significant bilateral silencing of up to 35% Htt mRNA [54]. hsiRNA^HTT^ alone, without packing into exosomes, did not show such an effect [54]. This can be a trajectory to use exosomes for delivery of the therapeutic oligonucleotides to treat NDs.

## 6. Conclusions

HD is a hereditary autosomal dominant neurodegenerative disorder. HD is incurable which represents a great challenge for patients and their families. Currently, there are several therapeutic interventions being developed to prevent or delay the onset of the symptoms and slow down the disease progression. However, the lack of powerful biomarkers that might be used to evaluate the efficiency of the applied therapies is still challenging.

Recently, there has been growing interest in extracellular vesicles (EVs), mainly exosomes, as a potential source for novel non-invasive biomarkers for NDs. Exosomes play significant roles in HDs and many other NDs by acting as a carrier of the misfolded proteins, and other molecules (RNAs and miRNAs) all over tissues and body fluids. Exosomes could be used both as a source of biomarkers to indicate the early stage of the disease and help understanding its pathogenicity, and as a therapeutic moiety, e.g., for delivery of gene silencing therapies. Due to their ability to cross the BBB and their availability in almost all body fluids, exosomes can be a potential source of novel, noninvasive biomarkers for HD, as well as many other diseases. Nonetheless, the use of exosomes as biomarkers in HD is still in the early stages and more studies need to be performed in this field. On the other hand, exosomes as possible carriers of novel therapies for HD and many other NDs are currently being investigated.

## Figures and Tables

**Table 1:** DNA- and RNA-based strategies targeting mHTT production pathway.

**Therapy**	**Mechanism**	**Delivery Method**	**Ref.**
**RNA-based therapies**			
**Antisense oligonucleotides**	Inhibit HTT mRNA and thereby reduce the concentration of mHTT, such as Tominersen (previously IONIS-HTTRx) *	Intrathecal	[51]
	Targeting the CAG repeats in HTT mRNA to reduce the mHTT level using ASO with the CUG-7 sequence	Intrathecal	[52]
**RNA interference (RNAi)**	Targeting HTT mRNA using hydrophobically-modified siRNAs (hsiRNA)	Direct uptake in cell culture	[53]
	Targeting HTT mRNA to reduce mHTT expression level using hsiRNA^HTT^	Exosomes	[54]
	Silence HTT mRNA using cholesterol-conjugated hsiRNAs	Exosomes	[55]
	Targeting human huntingtin exon 1 (HuHtt) transcript	Exosomes	[56]
	Targeting RE1-Silencing Transcription Factor (REST) using miR-124	Exosomes	[57]
	Induce HTT gene silencing by targeting the heterozygous single nucleotide polymorphism (SNP) rs362331 in exon 50 or rs362307 in exon 67 linked to mHTT using artificial miRNAs (miHTTs)	Adeno-associated virus sero type 5 (AAV5) vector (Bilateral injection)	[58]
	Targeting HTT mRNA which then reduces and prevents the neuronal dysfunction using an engineered miRNA	AAV5 (Intracranial)	[59]
	Targeting SNP (single nucleotide polymorphism) using artificial miRNA (miR-451) to silence the mHTT allele which then results in suppression of mHTT aggregation and prevents neuronal dysfunction	AAV5 (Intracerebral)	[60]
	Targeting exon 48 of the human HTT mRNA using artificial miRNA (miR-155)	Adeno-associated virus serotype 9 (AAV9) (Striatal and cortical injection)	[61]
**DNA-based therapies**			
**Zinc Finger Proteins (ZFPs)**	Targeting mHTT gene expression using synthetic transcription repressor by binding to the expanded CAG repeats in mHTT gene	Adeno-associated virus (AAV) (Intraventricular injection)	[62] [63]
**CRISPER-Cas9**	Genome editing by targeting SNPs to induce mHTT allele specific inactivation resulting in permanent inactivation of the HD mutation	Transfection to fibroblasts	[64]
	Genome editing by the deletion of polyQ domain of the mHTT gene	AAV (Intracranial)	[65]

* Tominersen dosing discontinued in Phase III clinical study in March 2021 [66].

## Data Availability

Not applicable.
